# Guidance and Best Practices for Reestablishment of Non-Emergent Care in Nuclear Cardiology Laboratories During the Coronavirus Disease 2019 (COVID-19) Pandemic: An Information Statement from ASNC, IAEA, and SNMMI

**DOI:** 10.2967/jnumed.120.251355

**Published:** 2021-03

**Authors:** Hicham Skali, Venkatesh L. Murthy, Diana Paez, Elisa M. Choi, Felix Y. J. Keng, McGhie A. Iain, Mouaz Al-Mallah, Roxana Campisi, Timothy M. Bateman, Ignasi Carrio, Rob Beanlands, Dennis A. Calnon, Vasken Dilsizian, Maurizio Dondi, Alessia Gimelli, Robert Pagnanelli, Donna M. Polk, Prem Soman, Andrew J. Einstein, Sharmila Dorbala, Randall C. Thompson

**Affiliations:** a Division of Nuclear Medicine and Molecular Imaging, Department of Radiology, Brigham and Women’s Hospital, Harvard Medical School, Boston, MA; b Cardiovascular Division, Department of Medicine, Brigham and Women’s Hospital, Harvard Medical School, Boston, MA; c Frankel Cardiovascular Center, University of Michigan, Ann Arbor, MI; d Nuclear Medicine and Diagnostic Imaging Section, Division of Human Health, International Atomic Energy Agency, Vienna, Austria; e Atrius Health, Somerville, MA; f National Heart Centre, Singapore, Singapore; g Saint Luke’s Mid America Heart Institute, University of Missouri-Kansas City, Kansas City, MO; h Houston Methodist DeBakey Heart & Vascular Center, Houston Methodist Hospital, Houston, TX; i Departments of Nuclear Medicine and Cardiovascular Imaging, Diagnostico Maipu, Department of Nuclear Medicine, Instituto Argentino de Diagnostico y Tratamiento, Buenos Aires, Argentina; j Universitat Autonoma de Barcelona, Barcelona, Spain; k Division of Cardiology, University of Ottawa Heart Institute, Ottawa, Canada; l OhioHealth Heart and Vascular Physicians, Columbus, OH; m Department of Diagnostic Radiology and Nuclear Medicine, University of Maryland School of Medicine, Baltimore, MD; n Fondazione Toscana Gabriele Monasterio, Pisa, Italy; o Department of Radiology, Duke University Health System, Durham, NC; p Division of Cardiology, Heart and Vascular Institute, University of Pittsburgh Medical Center, Pittsburgh, PA; and; q Department of Medicine, Cardiology Division, and Department of Radiology, Columbia University Irving Medical Center, New York-Presbyterian Hospital, New York, NY

## INTRODUCTION

The Coronavirus Disease 2019 (COVID-19) pandemic is affecting healthcare systems and resources around the world in an unprecedented manner. Based on recommendations from the numerous medical specialty societies and governmental agencies such as the United States Centers for Disease Control and Prevention^1^ and Centers for Medicare & Medicaid Services^2^, non-urgent procedures and tests were either canceled or postponed during the buildup to and decline from the pandemic’s peak. The American Society of Nuclear Cardiology (ASNC) and the Society of Nuclear Medicine and Molecular Imaging (SNMMI) issued an Information Statement^3^ with guidance for nuclear cardiology laboratories during the COVID-19 pandemic in late March 2020 that also recommended delaying all non-urgent studies. Reestablishment of non-emergent care^4^ in nuclear cardiology laboratories will require a careful approach to continue to mitigate the risk of viral transmission while also providing crucial cardiovascular assessments for our patients. The current document prepared by ASNC, SNMMI, the International Atomic Energy Agency (IAEA) and the Infectious Disease Society of America (ISDA) describes potential practices for this reestablishment, reflecting diverse settings from the United States and worldwide.

It is important to note that these recommendations are primarily based on expert opinion, not systematically tested, and may provide guidance to supplement guidelines, regulations and legislation as well as institutional infection control policies.^2^ A survey of the authors’ opinions regarding several issues outlined in this document is available in the online supplement (supplemental materials are available at http://jnm.snmjournals.org).

## GENERAL PRINCIPLES

### Timing of Re-Opening/Resuming Activities in Stress/Nuclear Cardiology Laboratory:

1. Timing should follow national, state and local regulations and recommendations.^4–6^
2. Healthcare facilities should have adequate equipment and personnel and not be in crisis management mode or diversion mode. For inpatient laboratories, there should be available hospital beds and intensive care unit beds.
3. In general, we recommend that utilization of nuclear cardiology services parallel opening of upstream and downstream resources such as clinics, catheterization laboratories, operating rooms and other services, so as to facilitate uniform care delivery.

###  COVID-19 Testing:

The diagnosis of COVID-19 is based on the detection and identification of the virus by means of the reverse transcription polymerase chain reaction (RT-PCR), a molecular diagnostic technique based on the detection of specific genetic sequences of the virus. Antibody tests detect immunoglobulin antibodies from an immune response to SARS-CoV-2 and are not appropriate for diagnosing current COVID-19 infection. Laboratories should rely on molecular tests to diagnose the presence of SARS-CoV-2 infections. However, a negative molecular test result does not rule out COVID-19 for several reasons. Some patients may have negative initial tests due to low viral load during the early days of infection or inadequate sampling of the posterior nasopharynx. Thus, social distancing and PPE are critical to prevention of laboratory transmission of COVID-19. When testing capacity allows, using a molecular test to test for COVID-19 infection prior to appointments in the nuclear cardiology laboratory is recommended combined with screening patients for a fever and symptoms consistent with COVID-19 infection upon arrival for testing appointments. Patients who are known to have an active infection with the COVID-19 virus should in the vast majority of cases have their nuclear cardiology test deferred.

###  Preventive Measures for COVID-19 Disease:

The implementation of preventive and mitigation measures is essential. The most effective preventive measures include performance of proper hand hygiene; avoidance of touching eyes, nose and mouth; practice respiratory hygiene when coughing or sneezing; wearing a medical mask and maintenance of physical distancing (ideally 2 meters or more).^8,9^

###  Personal Protective Equipment (PPE):

The goal of PPE use is to protect healthcare providers and patients and predominantly to minimize spread from asymptomatic individuals and surface exposure.^10^ Healthcare workers require additional precautions to protect themselves and prevent transmission in the healthcare environment. Precautions include wearing proper PPE and donning and doffing items properly. Recommendations for PPE use vary by institution, region and country and the type of PPE recommended will usually be based on institutional or regional policies. Currently most healthcare centers in the United States require universal use of a surgical mask by staff and visitors with selective use of more advanced PPEs. As nuclear cardiology and other laboratories re-expand their operations, a sufficient and increasing quantity of PPE will be required for protection for healthcare personnel and patients. PPE use is currently recommended throughout the nuclear laboratory when indicated; this use should follow national, state/regional, and local public health policies and recommendations.

## SPECIFIC PRINCIPLES FOR NUCLEAR CARDIOLOGY

###  Before the Test:

#### 1. Prioritization of a Study Request:

As we move into the next phase of the COVID-19 pandemic, in which newly diagnosed cases have declined in many regions, regulations are permitting flexibility and allow facilities to reinstate services for patients needing non-emergent non-COVID-19 care. Urgent patients should continue to be tested first and as quickly as prudently possible while ensuring patient and healthcare provider safety. It is important to remember that many of the patients with higher priorities are typically symptomatic whose risk of a cardiac event is not insignificant.

- For patients of equal urgency, those who have been waiting the longest for their procedure should be the first to undergo testing.
- Many patients have had testing delayed for several weeks during the period of sheltering in place; it is important for the provider team to maintain contact with patients and to encourage them to report changes in symptoms.
- Due to the delay in testing, extra caution needs to be exercised by clinical staff, including supervising physicians, as the patient’s clinical status may have deteriorated to the point where stress testing may no longer be safe or appropriate.
- For some patients requiring prior approval from the insurance carrier, the approval may have expired and need to be reinstated. However, some Radiology Benefit Management companies in the United States have recently extended the time period before expiration during the pandemic.
- Case prioritization can be performed primarily by laboratory professionals but is most commonly decided collectively and in collaboration with other colleagues from cardiology, surgery, and other disciplines.
- Early during the COVID-19 pandemic, many facilities prioritized patients being scheduled and those already scheduled according to the perceived clinical urgency of the study. Please see an example of a prioritization scheme using four categories in Table 1. Some laboratories use a 3-category scheme instead.^3^

**TABLE 1:** An Example of a Prioritization Scheme

Urgent inpatient or outpatient (to be performed upon request): Inpatient or outpatient with a clinical scenario suggesting a moderate to high likelihood of short-term major adverse cardiac events, in whom the results of testing would have high likelihood of modifying management
Higher priority (deferred for 1–2 months): Inpatient or outpatient who meets AUC criteria for testing, but with a clinical scenario suggesting a low likelihood for short-term major adverse cardiac events
Lower priority (deferred for 2–4 months): Outpatient, who meets AUC criteria for testing, and who is clinically stable, expected to have normal or low-risk findings that would not be expected to affect short-term management. Some examples are pre-operative testing for elective surgery; surveillance testing such as in asymptomatic patients with prior history of PCI or CABG, a patient already on class 1C antiarrhythmic, a patient post cardiac transplant; asymptomatic patient with an elevated coronary artery calcium score
Elective (deferred for 4–6 months): Screening or wellness tests such as coronary artery calcium scans and treadmill exercise tests

####  Scheduling a Patient:

Scheduling of patients should take into account a number of parameters including institutional and laboratory resources for safe performance of testing during the COVID-19 pandemic.

- Institutional policies: When increasing patient volume through the nuclear cardiology laboratory, it is also important that this be coordinated with the affiliated institution to ensure that service delivery is consistent with their overarching plan during the pandemic.
- Staff: Availability of laboratory staff is an equally important consideration, especially if many have been redeployed, furloughed or become ill as a result of the pandemic.
- Operating hours: As utilization increases, laboratories should consider extending to early/late hours and/or weekend testing to allow the backlog of patient tests to be completed and to allow more effective social distancing. This approach should be balanced with availability of staff and key equipment, especially PPE.
- Phased opening: A phased opening with a ramp up period is generally recommended by health agencies^2^. For example, 25% of a standard case load might be planned for the first 1–2 weeks, 50% case load in weeks 3 and 4, and then greater caseloads if conditions permit. Concomitant overlapping cases should be avoided to provide for appropriate social distancing.
- Adequacy of supplies: To reestablish operations it is necessary to ensure the availability of radioisotopes which are produced in a limited number of facilities worldwide. One of the effects of the pandemic has been the disruption of distribution channels, including international cargo flights. In addition, many radiopharmacies have either furloughed or laid off staff because of reduced demand over the period of sheltering in place during the pandemic.
- Resources: In addition, the laboratory needs to ensure that they have adequate resources to safely perform testing, keeping in mind that the demand for certain equipment will grow as other healthcare providers ramp up deferred services.
- Communication/patient concerns: A significant proportion of patients may decline testing because of concerns about the risk of contracting COVID-19 by visiting the testing facility. Institutions must provide referring physicians detailed information about steps taken by the laboratory to minimize the risk of COVID-19 infection. Referring providers must remind patients about the real and significant risk of not undergoing testing. Staff scheduling patients should have scripting available to screen patients for symptoms consistent with COVID-19 to reassure patients, including a description of the additional precautions that are being undertaken by the laboratory to minimize COVID-19 infection risk. If a patient refuses to undergo testing it is of the utmost importance for the nuclear cardiology laboratory to inform the referring physician to let them know of the patient’s decision so that they can contact the patient to discuss this further and make alternate arrangements.
- Tracking: Establishment of proper systems to track canceled or postponed studies is very important. Providers should establish a priority score that can include indication for testing and overall CV risk, and COVID-19 risk.

###  Day of Testing:

Specific precautions are recommended on the day of the test at the time of arrival, during the imaging test and during the stress test to minimize COVID-19 exposure risk as outlined below. An important principle is to minimize the amount of time spent by patients in the nuclear cardiology laboratory or at the associated institution to reduce risk both for patients and staff.

####  Arrival/Registration:

A key goal is to avoid overcrowding of the elevators, waiting rooms, corridors, and scanners while not delaying patient care. The test procedures need to allow for adequate social distancing between patients before and during the whole testing process.

- Appropriate physical distancing should be maintained at all stages of patient engagement, including registration, waiting areas, and consent. Waiting areas and processes should be arranged to allow appropriate physical distancing. In addition, accompanying visitors and family members should not be allowed or should be limited to one person and only if their presence is essential. If the facility has significant space constraints, consideration should be to having patients wait in their vehicles until contacted by cellphone by a member of the nuclear cardiology laboratory staff. The patient would then be met at the entrance of the facility by the staff member and escorted to the laboratory.
- The use of telehealth to assess patients for new symptoms, to register the patient, to provide them with details about the test to be performed, and to obtain verbal consent is generally recommended. Processes should also be automated as much as possible to reduce face to face interaction. Some onsite administrative steps can be performed through the patient’s smartphone. The healthcare screening questionnaire should be thorough.
- Temperature measurements and a screening questionnaire for all patients are generally recommended at arrival to healthcare facility and to nuclear laboratory.
- Face covering or masks are currently mandated at most medical centers (surgical/cloth/specific mask type as determined by local rules and infection control) for all patients, visitors, and healthcare personnel.
- Review indication for testing at registration or immediately upon a patient’s arrival in clinical areas to ensure that the test is still indicated and urgent. Changes in symptoms and/or health status may either render the test not indicated or demand a different test.

####  Imaging Considerations:

The overarching goals are to perform rapid and hygienic imaging to minimize COVID-19 exposure to healthcare professionals and patients.

Protocol selection
  •For SPECT myocardial perfusion imaging studies, elect stress first/stress only imaging when possible to potentially decrease duration of the study, and consider rapid acquisition protocols when feasible.
  •For SPECT myocardial perfusion imaging studies in inpatients who are not eligible for stress only testing, consider performing the rest injection in the patient’s inpatient room, to avoid completely or minimize waiting time in the laboratory. A typical protocol would begin with a technologist bringing a dose of technetium-99m sestamibi or tetrofosmin to the patient’s inpatient room, checking the intravenous line, and injecting the dose. After a suitable delay, the patient is transported to the laboratory, directly to a waiting open camera, where rest imaging is performed. After an appropriate delay, when the patient waits in their hospital room, he or she is brought to an open stress room where stress testing is performed. After stress testing, the patient is transported back to their room, and returns to a waiting open camera approximately 1–1.5 hours after stress injection.
  •For technetium-99m pyrophosphate (PYP) scans in inpatients, consider injecting the patient and allowing him/her to leave the laboratory, returning for imaging after the laboratory protocol delay.
  •For N-13 ammonia PET myocardial perfusion imaging studies, consider performing a protocol facilitating a single rest/stress session on the camera with enhanced social distancing, and avoid the patient leaving the camera room between rest and stress sessions and the camera needing cleaning multiple times. A low-dose rest, high-dose stress protocol may enable a more rapid single-session imaging.
Imaging time slots may need to be expanded and extra time should be allowed for cleaning rooms, cameras, chairs, and other surfaces between patients.
Appropriate cleaning of equipment between cases is essential.
For outpatients requiring rest imaging, consideration could also be given to performing this on a separate day to give greater control of workflow and minimize time within the department, although this approach may be less appropriate in some settings and needs to be balanced by the downside of using more PPE for 2-day imaging.
For laboratories with access to myocardial perfusion PET using Rb82, this modality is preferred because of its time efficiency. A complete rest-stress PET MPI study can be acquired within 30 to 45 minutes, not only minimizing exposure for patients and staff, but also potentially reducing number of rooms and spaces which could experience surface contamination and possibly reducing staffing needs.
For patients tested with SPECT-CT or PET-CT instrumentation in regions with higher prevalence of COVID-19 infections, some laboratories recommend a policy of reviewing the chest CT images before the patient leaves the laboratory. For patients who have lung infiltrates seen on chest CT images, they may require further evaluation for possible COVID-19 infection.^12^

####  Stress Laboratory Considerations:

The goals are to perform a safe test for the patient and minimize droplet exposure to healthcare professionals and patients.

- If possible, consider pharmacologic testing preferably using vasodilator stress agents to decrease droplet exposure risk,^9^ especially in patients who are not known to be COVID-19 negative. Patients breathing heavily during exercise may generate droplets and traditionally stress test personnel are in close proximity to patients. Pharmacologic testing is not inferior to exercise testing in terms of diagnostic performance. Regadenoson may be the preferred stress agent (if available and not contraindicated), since it requires a single 10 second infusion, after which providers can maintain a safe distance from the patient. For adenosine and dipyridamole stress testing, extra-long tubing can be used to keep distance between staff and patients.
- If after careful consideration, exercise testing is determined to be necessary, it is recommended to use a higher-level PPE for staff (for example N95/FFP2/P2/DS/KN95/Korea 1^st^ class respirator or equivalent, face shield, gloves and gown) and surgical mask or face covering for patients, assuming these supplies are available,^11^ and extra time should be allowed in order to clean the room.
- Appropriate cleaning between patients is required.

## ONGOING CONSIDERATIONS FOR THE LABORATORY

An important goal of an ongoing monitoring process is to refine policies and procedures as needed in response to the evolution of the COVID-19 pandemic as the laboratory operations resume to include more elective procedures.

A. Additional data collection and monitoring may be warranted. The nuclear cardiology laboratory’s protocols and patient scheduling templates will need to be closely monitored and refined multiple times over the coming months as the COVID-19 pandemic slowly recedes, with potential for local or widespread waves of new COVID-19 infections. Data collection and monitoring will be necessary to inform the need to modify existing laboratory policies. Some suggested parameters for collecting and monitoring and potential responsive changes to laboratory workflow are listed in Table 2.
B. Availability of laboratory personnel might be impacted by redeployment to other work responsibilities in the healthcare system during the COVID-19 pandemic, staff quarantine to home due to COVID-19 infection of the staff or their close contacts, and other non-COVID-19 related issues.
C. Laboratory testing hours should be adjusted as needed.
  - Expansion of testing might need to be suspended completely if essential laboratory personnel are unavailable. Similarly, if the supply of PPE or COVID-19 testing is interrupted, test scheduling would need to be reduced to allow for adequate supply of PPE and COVID-19 testing for other patient care areas of higher priority.
  - If, however, there is a large backlog of orders for nuclear cardiology procedures as a result of deferred testing during the height of the COVID-19 pandemic, laboratories might need to consider temporarily extending weekday hours of operation or weekend testing to accommodate a surge in patient volume. The feasibility of extending hours of operation will depend of the availability of laboratory personnel, PPE, and COVID-19 testing.

**TABLE 2:** Workflow Parameters to Monitor in the Nuclear Cardiology Laboratory

Parameter for monitoring	Impact on laboratory policies
Decreased laboratory personnel availability	Reduce hours of operation
Decreased availability of PPE and COVID-19 testing	Reduce hours of operation
Increased test duration (including cleaning)	Adjust the patient scheduling template
Increased or pending orders for cardiac imaging procedures	Increase hours of operation

D. Tracking of total time required for nuclear cardiology procedures is desirable. This includes time required to fully clean and disinfect the stress testing equipment and the imaging equipment. This information will inform patient scheduling templates.

## SAFETY AND RISK FOR POTENTIAL SECOND OR SUBSEQUENT WAVES

While one cannot predict the potential for a second or subsequent waves of COVID-19 pandemic, numerous public health authorities are warning that it is possible, and procedures need to be in place to mitigate the adverse effects of a second wave. Hence, it is important to follow the steps below:

- Providers and institutions should monitor local data and follow national, state, and department of public health recommendations for possible second COVID-19 waves that may require decreasing nuclear cardiology laboratory activities and enhanced protective measures.
- Physical distancing and masking should be maintained for the foreseeable future.
- Limitations on visitors and accompanying persons should be continued for the foreseeable future as well.

## NUCLEAR CARDIOLOGY TRAINING AND EDUCATION

Trainees are an important part of nuclear cardiology programs and laboratories. During the COVID-19 pandemic, many trainees have been redeployed from their imaging rotations to clinical care of COVID-19 patients in the hospital and intensive care units. If the pandemic continues, trainee education needs to be revised to use novel training methods including web and video-based approaches to learning. New educational procedures need to be consistent with policies from stakeholders such as the Accreditation Council for Graduate Medical Education, American Board of Internal Medicine, American Board of Nuclear Medicine, American Board of Radiology, and Nuclear Regulatory Commission. This is particularly pertinent for hands-on education in the stress laboratory, hot laboratory, and radiopharmacy, which may need to be adapted to video methods. The number of cases required for graduation may need to include a larger percentage of virtual educational sessions, in compliance/collaboration with certifying agencies including the Certification Board of Nuclear Cardiology, American Board of Internal Medicine, the American Board of Nuclear Medicine, and the American Board or Radiology.

## ADDITIONAL CONSIDERATIONS

- Staff should be monitored for wellbeing during these sometimes-stressful times, especially if required to work long hours and if they are redeployed to unfamiliar areas.
- In the United States, nuclear cardiology services are often impacted by the details of the patient’s health insurance policy and enforcement by radiology benefits managers. More advocacy effort will be required to convince these oversight agencies to allow relaxation of previous policies on prior authorization and flexibility for longer approval windows and to resist one-size-fits-all prescribed approaches.
- Laboratory leadership should consider redeployment of physician and nursing staff away from routine (elective) office activities until backlog has been reduced, especially for higher priority patients. This approach will help get the higher risk patients whose tests have been deferred completed, while not adding more and more higher priority patients to the schedule.
- Nuclear cardiology laboratory operations, of course, are part of the local healthcare systems and work in conjunction with other departments in the hospital and areas of the medical practice. Most of the suggestions outlined above must be instituted in concert and collaboration with other key departments.

## CONCLUSIONS

The unprecedented COVID-19 pandemic has disrupted healthcare systems, including nuclear cardiology laboratories around the world, severely reducing their services. As the health crisis recedes, nuclear cardiology laboratories are able to gradually expand operations while maintaining appropriate safe practices. This document lays out guidance about best practices, but it is important to recognize that conditions are quite different in various locations and will change in the coming months. An individualized, adaptable, and thoughtful approach that coordinates with other providers and public health authorities will be needed for the foreseeable future.

## DISCLOSURES

Dr. Bateman: Activities related to the present article: No relevant relationships. Activities not related to the present article: Research Grants: Bracco, GE Healthcare, Jubilant DraxImage. Consultant: AIM, AstraZeneca, Curium, GEHC. Royalties: SPECT and PET software products. Ownership interest: CVIT. Dr. Beanlands: Activities related to the present article: No relevant relationships. Activities not related to the present article: Grants/Honoraria from Jubilant DraxImage, LMI, GE Healthcare. Dr. Dorbala: Activities related to the present article: No relevant relationships. Activities not related to the present article: Grants and Honoraria from Pfizer and GE Healthcare. Institution has grants/grants pending from National Institutes of Health and International Atomic Energy Agency. Dr. Einstein: Activities related to the present article: No relevant relationships. Activities not related to the present article: Consultant for GE Healthcare and W. L. Gore and Associates; institution has grants/grants pending from National Institutes of Health, International Atomic Energy Agency, Canon Medical Systems, Roche Medical Systems, and W. L. Gore and Associates; received travel/accommodations/meeting expenses unrelated to activities listed from HeartFlow. Dr. Murthy: Activities related to the present article: No relevant relationships. Activities not related to the present article: Owns stock in General Electric and Cardinal Health and stock options in Ionetix. He has served as a paid advisor to Ionetix and Covidien. He has received research grants from Siemens and non-financial research support from INVIA Medical Imaging Solutions. He has received payment on behalf of Jubilant Draximage for expert witness testimony. Dr. Soman: Activities related to the present article: No relevant relationships. Activities not related to the present article: Serves on the advisory board of Alnylam Pharmaceuticals and is the principal investigator of a grant from Astellas Pharmaceuticals to the University of Pittsburgh. The remaining authors have no relevant relationships.
